# Antibacterial toxin colicin N and phage protein G3p compete with TolB for a binding site on TolA

**DOI:** 10.1099/mic.0.000024

**Published:** 2015-03

**Authors:** Helen Ridley, Jeremy H. Lakey

**Affiliations:** Centre for Bacterial Cell Biology, Institute for Cell and Molecular Biosciences, Newcastle University, Framlington Place, Newcastle upon Tyne NE2 4HH, UK

## Abstract

Most colicins kill *Escherichia coli* cells by membrane pore formation or nuclease activity and, superficially, the mechanisms are similar: receptor binding, translocon recruitment, periplasmic receptor binding and membrane insertion. However, in detail, they employ a wide variety of molecular interactions that reveal a high degree of evolutionary diversification. Group A colicins bind to members of the TolQRAB complex in the periplasm and heterotrimeric complexes of colicin–TolA–TolB have been observed for both ColA and ColE9. ColN, the smallest and simplest pore-forming colicin, binds only to TolA and we show here that it uses the binding site normally used by TolB, effectively preventing formation of the larger complex used by other colicins. ColN binding to TolA was by β-strand addition with a *K*_D_ of 1 µM compared with 40 µM for the TolA–TolB interaction. The β-strand addition and ColN activity could be abolished by single proline point mutations in TolA, which each removed one backbone hydrogen bond. By also blocking TolA–TolB binding these point mutations conferred a complete *tol* phenotype which destabilized the outer membrane, prevented both ColA and ColE9 activity, and abolished phage protein binding to TolA. These are the only point mutations known to have such pleiotropic effects and showed that the TolA–TolB β-strand addition is essential for Tol function. The formation of this simple binary ColN–TolA complex provided yet more evidence of a distinct translocation route for ColN and may help to explain the unique toxicity of its N-terminal domain.

## Introduction

Colicins are plasmid-encoded bacteriocins secreted by *Escherichia coli* which kill closely related competing bacteria by penetrating their outer membranes and delivering a toxic domain into or beyond the inner membrane (Cascales *et al.*, 2007). Initially, they bind a cell surface receptor, after which they recruit a translocator protein to cross the outer membrane. Colicins are classified into Group A or B according to the periplasmic proteins they use for translocation. Group B uses the Ton system, whilst Group A utilizes the Tol proteins Q, R, A and B, and includes ColA, ColN, ColE1–9 and ColK (Kim *et al.*, 2014). TolA is a 421-residue protein (Levengood & Webster, 1989) consisting of three domains: TolA_I_, TolA_II_ and TolA_III_. TolA_I_ is a transmembrane helix, anchored to the inner membrane, which interacts with TolQ and TolR (Derouiche *et al.*, 1995), whilst TolA_II_ is a 250-residue helical region with an opaque role in colicin function (Schendel *et al.*, 1997). TolA_III_ is involved in a range of protein–protein interactions (Kim *et al.*, 2014). TolA has an important role in outer membrane integrity (Fognini-Lefebvre *et al.*, 1987; Webster, 1991) and is also involved in the import of filamentous bacteriophage DNA (Click & Webster, 1998; Webster, 1991). TolB is a 47 kDa two-domain soluble protein which interacts both with TolA_III_ and via a high-affinity interaction with the outer-membrane-bound, peptidoglycan-associated lipoprotein Pal (Abergel *et al.*, 1999; Bonsor *et al.*, 2009; Bouveret *et al.*, 1995). ΔTol mutants are able to replicate, but members of the Tol complex move to the central division zone during cell division, implying a non-essential role in this process (Gerding *et al.*, 2007).

One emerging characteristic of colicin biology is that although the general mechanisms are similar, there is an astonishing variation in the specific molecular interactions employed by different colicins (Grinter *et al.*, 2014; Jakes, 2014; Kleanthous, 2010). ColA and ColN are Group A pore-forming colicins and cell killing is via formation of an ion channel in the inner membrane (Baty *et al.*, 1990). ColA and ColN comprise three distinct domains having roles in each of the three insertion steps. ColN, uniquely, uses LPS as its primary receptor on the cell surface and binds to it with its central receptor-binding domain ColN-R (ColN_91–183_) (Johnson *et al.*, 2014). Like ColE9 (Housden *et al.*, 2010, 2013) it also binds to outer membrane protein F (OmpF) via an OmpF-binding site at the extreme N terminus of its disordered translocation domain (T domain), ColN-T (ColN_1–90_) (Johnson *et al.*, 2013). It then binds to TolA_III_ (Anderluh *et al.*, 2003) via a TolA-binding site (TABS) in the central region of ColN-T (ColN_40–67_). Like most other colicins, ColA-R (ColA_173–388_) binds to a protein receptor, the vitamin B_12_ uptake protein, BtuB, as its primary receptor, and then recruits OmpF before ColA-T (ColA_1–172_) binds to both TolA and TolB (Bouveret *et al.*, 1998; Kim *et al.*, 2014). The pore-forming domains are sited at the C-terminal end. In this study of TolA binding we were thus mainly interested in the colicins’ N-terminal T domains which contain intrinsically disordered regions.

A high-resolution X-ray structure of ColA-T_53–107_ in complex with TolA_329–421_ is available (Li *et al.*, 2012) and the TolA-binding ‘box’ of ColN (ColN_40–67_) has been identified by mutagenesis, NMR and bioinformatics (Anderluh *et al.*, 2003, 2004; Gokce *et al.*, 2000; Hecht *et al.*, 2008, 2009; Raggett *et al.*, 1998). The TolQRAB complex interacts with other proteins of the outer membrane and inner leaflet such as Pal, Lpp and OmpA, establishing an energy-dependent link between the inner and outer membranes (Cascales *et al.*, 2000). As well as interactions with colicin T domains, TolA_III_ is also known to bind the N-terminal (N1) domain of G3p (gene-3-protein) – a minor coat protein located at the end of the phage capsid of filamentous bacteriophage (Click & Webster, 1997; Riechmann & Holliger, 1997). A high-resolution structure of TolA_295–421_ in complex with G3p-N1_1–86_ has been described by X-ray crystallography [Protein Data Bank (PDB) ID: 1Tol] (Lubkowski *et al.*, 1999) and NMR (PDB ID: 1S62) (Deprez *et al.*, 2005). In the native structure of G3p-N1-N2 (PDB ID: 2g3p) (Holliger *et al.*, 1999), the N1 domain binds to the N2 domain via ~30-residue contacts and TolA_III_ was found to interact with the same region (Lubkowski *et al.*, 1999). This situation is mirrored in ColN where the ColN-T TABS epitope binds to both ColN-R and TolA_III_ (Hecht *et al.*, 2008). Both ColN-T and G3p-N1 bind TolA_III_ with similar affinity, *K*_D_~1 µM (Anderluh *et al.*, 2004; Gokce *et al.*, 2000; Karlsson *et al.*, 2003; Raggett *et al.*, 1998).

The crystal structure of ColN has been resolved by X-ray crystallography to 3.1 Å resolution (Vetter *et al.*, 1998); however, the T domain, a highly dynamic region, was unresolved. It was later shown by NMR that the disordered ColN-T is not entirely unstructured but, in an analogous way to G3p, ColN-T TABS is associated with the rest of Col-N (Hecht *et al.*, 2008). Furthermore, it was shown that ColN-T TABS binds to similar regions of TolA_III_ as G3p-N1 (Hecht *et al.*, 2009). Here, we used mutagenesis to map the ColN-T-binding site of TolA_III_. Mutants were designed using the current X-ray structures of TolA_III_ in complex with G3p-N1 (Lubkowski *et al.*, 1999) and ColA-T (Li *et al.*, 2012), and previous NMR studies. These mutants were analysed both *in vitro* using surface plasmon resonance (SPR) and *in vivo* using whole-cell killing assays to demonstrate a direct link between protein–protein interactions and toxicity for each mutant.

## Methods

### 

#### Bacterial strains, plasmids and protein purification.

See supplementary methods available in the online Supplementary Material.

#### Spot test killing assay.

The activities of ColN and ColA were assayed using the established spot test dilution assay (Pugsley & Schnaitman, 1978). Dilutions of colicin were spotted onto a lawn of JC207 (ΔTolA) *E. coli* cells harbouring the TolA-encoding WT pSKL10 or mutant plasmids and incubated at 37 °C for 16 h. The degree of complementation of the TolA deletion was taken as the lowest concentration of colicin that produced a zone of clearance.

#### Liquid culture killing assay.

A single colony of *E. coli* JC207 cells carrying mutant or WT pSKL10 plasmid was inoculated into 5 ml lysogeny broth (LB) containing 100 µg ampicillin ml^−1^ [LB(amp)] and grown overnight at 37 °C. Cells were diluted to OD_600_ 1.7 with LB(amp) and 5 µl added to each well of a 96-well flat-bottomed microtitre plate containing 135 µl LB(amp) (pre-warmed to 37 °C). These were grown at 30 °C, with 4 mm double-orbital shaking at 150 r.p.m. in a FluoStar Optima plate reader, 60 cycles of 600 s with 20 flashes per cycle, until OD_600_~0.4 was reached (cycle 26, 273 min). Then, 15 µl 100 nM ColN (in LB medium) was added. This gave a final ColN concentration of 10 nM, which was above the MIC of 0.5–1 nM (Sharma *et al.*, 2009). Blank wells containing LB(amp) only were subtracted from the data. Growth curves were plotted using SigmaPlot software and represent the means of two separate sets of three wells. The final OD_600_ was taken at cycle 42 (153 min after colicin addition) and was used to calculate the percentage killing of ColN (see Table 1) with data normalized to 0 % killing for cells only and 100 % killing for cells complemented with WT pSKL10.

**Table 1.** Strains and plasmids used to express TolA and mutants Complementation data represent the lowest concentration able to kill JC207(ΔTolA) plus that plasmid on the plate assay; >10 000 nM means no killing observed at any concentration. The percentage killing by ColN in liquid culture was normalized to 0 % killing for JC207 cells only and 100 % killing for JC207 cells complemented with WT pSKL10. The α-helix content percentage of TolA_III296–421_ was given by the signal magnitude at 222 nm compared with WT ( = 100 %).

Strain	TolA_III_ mutation	Complementation (nM)		ColN killing (%)	α-Helix in far-UV CD (%)
		ColN	ColA
JC207	ΔTolA	>10 000	>10 000	0	–
JC207 (pSKL10)	WT TolA_III_	100	500	100	100
JC207 (PUC19)	ΔTolA (negative control)	>10 000	>10 000	14	–
JC207 (pSKL10G334A)	G334A	100	500	88	–
JC207 (pSKL10D336A)	D336A	100	500	100	–
JC207 (pSKL10I337A)	I337A	100	500	>100	–
JC207 (pSKL10N338G)	N338G	100	500	91	–
JC207 (pSKL10N339A)	N339A	100	1000	76	–
JC207 (pSKL10Y340A)	Y340A	5000	1000	29	66
JC207 (pSKL10A341F)	A341F	100	500	59	–
JC207 (pSKL10A341R)	A341R	500	500	41	–
JC207 (pSKL10G342E)	G342E	100	500	66	89
JC207 (pSKL10Q343A)	Q343A	100	500	63	–
JC207 (pSKL10I344D)	I344D	>10 000	>10 000	0	46
JC207 (pSKL10K345A)	K345A	100	500	>100	–
JC207 (pSKL10S346A)	S346A	100	500	96	100
JC207 (pSKL10I348D)	I348D	>10 000	>10 000	14	60
JC207 (pSKL10E349A)	E349A	100	500	92	–
JC207 (pSKL10 I367A)	I367A	500	1000	81	54
JC207 (pSKL10 L369A)	L369A	500	1000	69	55
JC207 (pSKL10E381A)	E381A	500	1000	92	82
JC207 (pSKL10A391E)	A391E	–	–	–	29
JC207 (pSKL10F412A)	F412A	1000	1000	39	–
JC207 (pSKL10A415R)	A415R	100	500	76	–
JC207 (pSKL10A415L)	A415L	100	500	>100	–
JC207 (pSKL10P416E)	P416E	500	500	75	–
JC207 (pSKL10D418P)	D418P	>10 000	0	<0	100
JC207 (pSKL10D418V)	D418V	100	500	80	116
JC207 (PSKL10F419A)	F419A	1000	500	45	58
JC207 (pSKL10K420P)	K420P	>10 000	10 000	<0	93

#### SDS sensitivity assay.

Using a method similar to that described previously (Walburger *et al.*, 2002), JC207 ΔTolA cells harbouring pSKL10 (WT or mutant) plasmid or a pUC19 vector control were grown to OD_600_ 0.5 at 37 °C and then diluted 100-fold into LB(amp) (pre-warmed to 37 °C) supplemented with 0 (LB added), 0.1, 0.5 or 1 % SDS (w/v in LB) and grown at 30 °C. The percentage of surviving cells was calculated from the OD_600_ after 180 min growth from the OD_600_ of the control pUC19 vector at 0 (100 % survival) and 180 min (0 % survival).

#### Alkaline phosphatase activity.

We used the method described by Torriani (1966). Briefly, cells were cultured in minimal Tris medium (pH 7.4). Single colonies of JC207 periplasmic-leaky cells and cells complemented with WT TolA (pSKL10) or TolA mutant plasmids were inoculated into 5 ml medium and grown for 8 h at 37 °C. An aliquot of 1 ml was inoculated into 50 ml medium and grown overnight at 37 °C, 180 r.p.m. to a final OD_600_~2.5. Cells (1 ml) were centrifuged at 14 000 r.p.m. for 10 min to recover the extracellular extract. Then, 600 µl was mixed with 400 µl *p*-nitrophenylphosphate substrate (0.2 mg ml^−1^ in 1 M Tris, pH 8 buffer). After a 10 min incubation, alkaline phosphatase activity was measured by *A*_410_.

#### Circular dichroism (CDS) spectroscopy

##### Far-UV CD (250–185 nm).

Samples were exchanged into 20 mM sodium phosphate (pH 7.0) buffer using PD10 columns (GE Healthcare) as per the manufacturer’s protocol and concentrations were typically 0.5–0.7 mg ml^−1^. Spectra were recorded in a 0.2 mm path-length demountable cuvette using a Jasco-810 spectropolarimeter at 25 °C. Scans (buffer baseline performed under identical conditions subtracted) are given as the differential mean residue extinction coefficient Δϵ. The signal at 222 nm is an approximate measure of the α-helical content (Chen & Yang, 1971).

##### Near-UV CD (320–250 nm).

Samples, in the same buffer, were typically 0.5 mg ml^−1^. Spectra were recorded in a 1 cm path-length cuvette at 25 °C. Results are given as the molar ellipticity.

##### Thermal denaturation.

Samples in 20 mM sodium phosphate (pH 7) buffer at a concentration of ~0.15 mg ml^−1^ were monitored (in a 1 mm path-length cuvette) at 222 nm from 20 to 90 °C for TolA_III_ constructs and at 288 nm from 25 to 95 °C for G3p-N1 constructs. The fraction of TolA_III_ unfolded (*f*_u_) was calculated at *x* °C using:

$$f_{\text{u}}=\frac{V_{\text{x}}-V_{\text{u}}}{V_{\text{n}}-V_{\text{u}}}$$

where *V*_u_ represents the far-UV CD value at 222 nm for unfolded TolA_III_ (at 90 °C), *V*_n_ represents the far-UV CD value at 222 nm for native TolA_III_ (at 20 °C) and *V_x_* represents the far-UV CD value at 222 nm for TolA_III_ at *x* °C. Data were plotted using SigmaPlot software and *T*_m_ values derived from sigmoidal fits.

#### Binding studies: SPR.

Experiments were performed using CM5 sensor chips using a Biacore X-100 (GE Healthcare) at 25 °C. The running buffer was HEPES-EP (10 mM HEPES, pH 7.4, 150 mM NaCl, 3.4 mM EDTA and 0.05 %, v/v, P20 detergent). All immobilizations were performed at 5 µl min^−1^ using standard amine-coupling carbodiimide chemistry procedures with the ligand proteins at 10 µg ml^−1^ in 10 mM sodium acetate (pH 4.5) buffer. TolA_III_, TolA_III_ K420P, TolA_III_ D418P and G3p-N1 V44P were immobilized at 596, 564, 550 and 423 resonance units (RU), respectively, where 1 RU~1 pg protein mm^−2^ on the surface. For ColN-T binding to TolA_III_, an improved model fit and more accurate *K*_D_ were obtained by using a lower immobilization level of 155 RU TolA_III_ to eliminate mass transport effects. In all cases, an activated/blocked flow cell 1 surface was used as the control and subtracted from the data. Analyte proteins were exchanged into HEPES-EP buffer at concentrations described in the text. Binding kinetics were performed at 30 µl min^−1^ using the Kinetics Wizard (Biacore X-100 control software) and binding analyses were performed at 10 or 30 µl min^−1^ using manual mode. Surfaces were regenerated by washing with 10 mM glycine (pH 1.8) buffer. Binding models were fitted using BIAevaluation software (v2.0.1) to simultaneously obtain association *k*_a_ and dissociation *k*_d_ rate constants, and the equilibrium constant *K*_D._ *K*_D_ was also determined by applying a steady-state affinity model.

## Results

### ColN-T_1–90_ secondary structure predictions

**Fig. 1.**
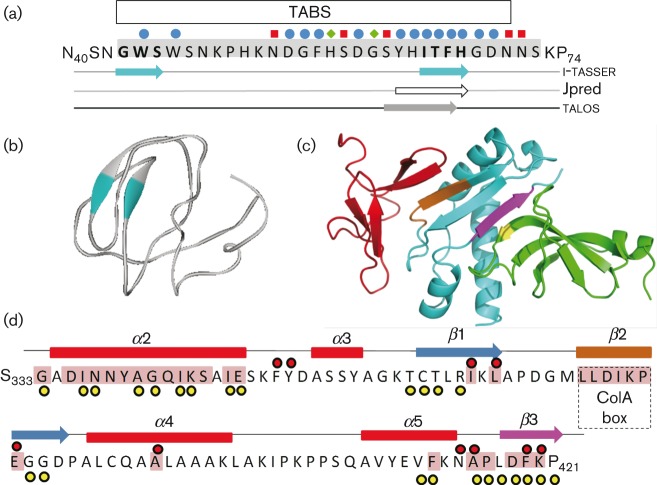
(a) Secondary structure analysis of ColN-T_1–90_. Structural predictions. Primary sequence of the ColN-T TABS, residues 40–74; there was no prediction of structure elsewhere. Previously reported binding to TolA_III_ (Anderluh *et al.*, 2004; Gokce *et al.*, 2000; Raggett *et al.*, 1998) is shown as WT binding (<10 µM) (red squares), intermediate binding (green diamonds) and no binding (blue circles). The self-recognition region determined by NMR by Hecht *et al.* (2008) is highlighted in grey. Schematic representation of the secondary structure predictions by the i-tasser three-dimensional model (Zhang, 2008) (top line), Jpred algorithm (Cole *et al.*, 2008) (middle line) and talos prediction (Cornilescu *et al.*, 1999) of the β-structure using the backbone chemical shifts of the T domain bound to TolA_III_ (bottom line). (b) i-tasser ribbon model of ColN-T_1–90_; coloured regions as in (a). (c) Binding of ColA-T (red) and G3p-N1 (green) to TolA_III_. Drawn in PyMOL (http://www.pymol.org/) using PDB ID: 3QDR and PDB ID: 1Tol, respectively. The ColA and G3p β-strand binding regions are shown in brown and magenta, respectively. Site of G3p-N1 V44P mutation shown in yellow. (d) Primary structure of TolA_III_ S333–P421. Secondary structure elements: α-helices (red blocks) and β-strands (blue arrows). Contacts with G3p-N1, inter-atomic distances <4.5 Å (yellow dots) (PDB ID: 1Tol; Lubkowski *et al.*, 1999). Residues which showed the strongest NMR chemical shift variations (>0.55 p.p.m.) upon binding ColN-T_1–90_ (Hecht *et al.*, 2009) are shown as red dots. The ColA-binding box (Li *et al.*, 2012) is shown. The residues mutated in this study are shaded pink. The ColA (β2) and G3p (β3) β-strand binding regions are shown in brown and magenta, respectively.

High-resolution structures of TolA_III_ in complex with ColA and filamentous bacteriophage M13 G3p proteins (Li *et al.*, 2012; Lubkowski *et al.*, 1999) show that binding occurs through β-strand addition such that a β-strand from one binding partner associates with a β-strand from the other, forming an extended β-sheet structure (Remaut & Waksman, 2006). NMR measurements showed that ColN-T TABS_40–67_ folds upon binding TolA_III_ (Anderluh *et al.*, 2003) and analysis of the amino sequence by the program pondr (Romero *et al.*, 1997) predicted that this region has a high probability of being ordered (Hecht *et al.*, 2008). Thus, it seemed likely that ColN-T TABS forms at least one new β-strand in order to bind TolA; to investigate this possibility we used the structural prediction algorithms i-tasser (Roy *et al.*, 2010; Zhang, 2008), talos (Cornilescu *et al.*, 1999) and Jpred (Cole *et al.*, 2008). An i-tasser model of T_1–90_ predicted two β-strands within the TABS (Fig. 1a, b). Although the *C* score of the model is low (−3.88), it is supported by talos and Jpred algorithm results in the S61–H67 region (Fig. 1a). Previous alanine-scanning mutant studies using SPR, isothermal titration calorimetry (ITC) and fluorescence spectroscopy showed that two regions, W44–W46 and Y62–F66, are essential for binding TolA_III_ (Anderluh *et al.*, 2003; Gokce *et al.*, 2000) (Fig. 1a) Furthermore, we have previously shown that talos analysis of ^1^H–^15^N NMR backbone chemical shifts (Cα, Cβ, CO and N) of ColN_40–76_ bound to TolA_III_ predicted ColN-T residues S61–F66 (SYHITF) to fall within the β-region of the Ramachandran plot (Hecht *et al.*, 2009) (Fig. 1a).

The structures of the ColA-T and G3p complexes are shown superimposed in Fig. 1(c), and the secondary structure of TolA_III_ is represented in Fig. 1(c) (taken from PDB ID: 1Tol; Lubkowski *et al.*, 1999). Although we used TolA_III296–421_ in this study, only residues 333–421 are shown in Fig. 1(d) as aa 296–332 are not resolved in any published structure or thought to be involved in interactions, and therefore were not mutated in this study. Structural contacts with G3p-N1 are indicated by yellow circles and the ColA-binding site (Li *et al.*, 2012) is shown (Fig. 1d). Residues which previously showed the strongest NMR chemical shift variations (>0.55 p.p.m.) upon binding ColN-T_1–90_ (Hecht *et al.*, 2009) are indicated by red circles and the residues mutated in this study are shaded pink (Fig. 1d).

### Defining ColN-T–TolA_III_ by mutagenesis

We initially selected mutation sites by studying the contacts in the crystal structures of TolA_III_ complexed with G3p-N1 and ColA-T. The TolA_III_ G3p (PDB ID: 1Tol; Lubkowski *et al.*, 1999) complex shows binding to TolA_III_ via two distinct regions: the first α-helix of TolA_III_ (α2 of full-length TolA) and β-strand addition to β3 (Fig. 1c). Mutants were created in these two regions. Mutations in α2 were: G334A, D336A, I337A, N338G, N339A, Y340A, A341F/R, G342E, Q343A, I344D, K345A, S346A, I348D and E349A; mutations in β3 were: F412A, A415L/R, P416L, D418P/V, F419A and K420P (Fig. 1d). Weitzel & Larsen (2008) have shown that an *E. coli* ΔTolA strain can be partially complemented by *Yersinia enterocolitica* TolA, regaining sensitivity to ColA, ColK and ColE1, but not ColN. Sequence alignment of the two TolA proteins revealed a non-conserved region within TolA α2 and the *E. coli* sequence GADINNYA was mutated to AGDISGYL to give a ‘*Y. enterocolitica*-like’ TolA. A ColA TABS mutant, 375-LLDIKP-380 to AAAAKA, as described by Li *et al.* (2012), was also constructed. Finally, we targeted residues with the strongest NMR chemical shift variations (>0.55 p.p.m.) upon binding ColN-T_40–76_ (Hecht *et al.*, 2009). This resulted in the following mutations: I367A, L369A, E381A, A391E, A415L and F419A.

### Colicin resistance of TolA mutants

**Fig. 2.**
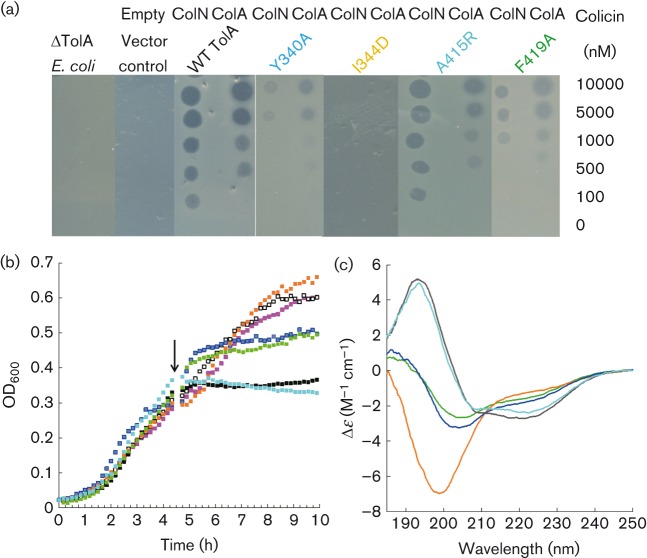
The effect of mutations in TolA_III_ on protein structure and colicin sensitivity. (a) A selection of spot test assays showing the colicin sensitivity of TolA mutants compared with WT pSKL10, an empty pUC19 vector control and ΔTolA *E. coli* cells. Concentrations of 10, 5, 1, 0.5, 0.1 and 0 µM ColN (left) or ColA (right) (in 50 mM sodium phosphate, pH 7.5, 300 mM NaCl) were spotted (2 µl) onto a lawn of *E. coli* JC207 ΔTolA cells complemented with pSKL10 WT TolA or mutant plasmids. (b) A selection of growth curves at 30 °C of JC207 ΔTolA cells complemented with pSKL10 WT or mutant TolA plasmid. Data are shown as WT TolA_III_ (black), pUC19 (negative control, white), JC207 cells only (magenta), Y340A (blue), I344D (orange), A415R (cyan) and F419A (green). A 10 nM final concentration of ColN was added after 273 min (arrow); growth continued for 153 min after which time the OD_600_ was used to calculate the percentage of killing using TolA_III_ WT and JC207 cells only to set 100 and 0 % killing, respectively (see Table 1). (c) A representative selection of far-UV CD spectra of TolA_III_ variants, measured in a 0.2 mm path-length cuvette at 25 °C. The protein concentration was typically 40 µM in 20 mM sodium phosphate, pH 7. Data are shown as WT TolA_III_ (black), Y340A (blue), I344D (orange), A415R (cyan) and F419A (green).

pSKL10, a plasmid expressing TolA, successfully complemented *E. coli* JC207 ΔTolA cells in both liquid culture and spot test assays, restoring both resistance to SDS and sensitivity to ColA and ColN. The selected mutations were made within the TolA_III_ region of pSKL10 and used to complement ΔTolA *E. coli* cells. Fig. 2 shows a selection of spot test assays and growth curves representing mutants displaying a WT TolA phenotype (e.g. A415R, cyan), partial phenotype (Y340A, blue; F419A, green) and complete resistance to colicin (I344D, orange) (see Fig. S1 for the full set of mutants). For spot test assays, the degree of complementation conferred by pSKL10 mutant plasmids to ΔTolA *E. coli* cells was measured as the lowest concentration of colicin that resulted in a zone of clearance on agar plates (Figs 2 and S1) and is given in Table 1. The resistance to both ColN and ColA was measured, and compared with WT pSKL10 complemented (TolA positive control) and non-complemented JC207 cells (TolA negative control). For the WT pSKL10 plasmid these values were 100 and 500 nM for ColN and ColA, respectively. Most mutations within the α2 region and those highlighted as targets by NMR showed WT-like activity. Only more structurally disrupting mutations, such as I344D and I348D (Figs 2 and S1), showed complete resistance (>10 000 nM) to both colicins. Growth curves were measured in liquid culture to investigate the effect of ColN in real-time. Again, most mutations displayed a WT colicin-sensitive TolA phenotype, with I344D and I348D showing complete resistance, and Y340A showing a partially resistant phenotype. The ‘*Y. enterocolitica*’ TolA chimera displayed a slightly greater resistance to ColN (500 nM) than WT (100 nM), but confirmed that α2 was not a critical site for ColN (Fig. S2). The ColA TABS mutant was resistant to ColA, but sensitive to ColN, indicating that the two colicins bound to TolA at different sites (Fig. S2).

### Structural characterization of TolA_III_ mutants

We made the same mutations in a plasmid that expressed only TolA_III296–421_, a well-characterized soluble domain truncate lacking the long helices of TolA_I–II_, and used far-UV CD spectroscopy to measure the secondary structure content. A selection of spectra is presented in Fig. 2(c). The WT TolA_III_ gave a typical αβ spectrum characterized by minima at 222 and 208 nm, and was comparable with previously published data (Anderluh *et al.*, 2004; Derouiche *et al.*, 1999). I344D and I348D (data not shown) showed spectra typical of an unfolded structure with negative maxima <200 nm, which explained their inability to complement the ΔTolA mutation. Some mutations did not affect the structure, whilst others showed limited loss of α-helical content compared with the WT protein (Fig. 2c; Table 1). We believe this was due to a partial or full collapse of the α2 or α4 helix.

### Testing the hypothesis that ColN-T binds TolA_III_ via β-strand complementation at β3

**Fig. 3.**
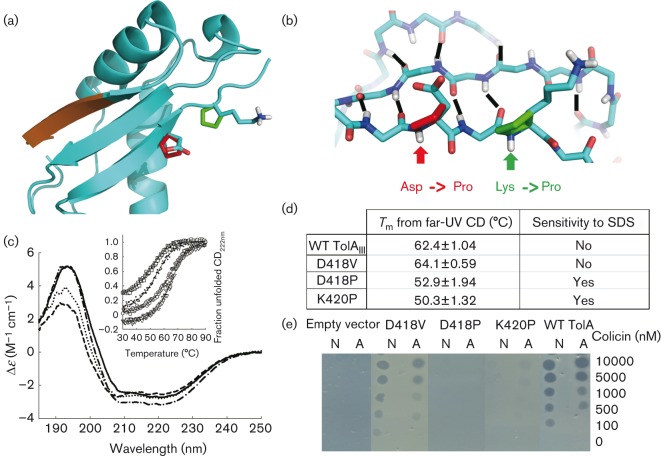
Mutations in TolA_III_. (a) Cartoon representation of the TolA_III_ domain showing sites of proline insertion in β3 (D418P, red; K420P, green). Orange region shows the ColA-binding site from Li *et al.* (2012). (b) Backbone representation of β1–3 showing mutation sites and internal hydrogen bonding. Backbone amide groups removed by proline mutations are highlighted. (c) Far-UV CD spectra of TolA_III_ variants. CD spectra were measured in a 0.2 mm path-length cuvette at 25 °C. The protein concentration was typically 43 µM in 20 mM sodium phosphate, pH 7. Data are shown as WT TolA_III_ (solid line), D418P (dotted line), D418V (dot-dashed line) and K420P (dashed line). Inset: normalized far-UV CD (222 nm) thermal denaturation profile (1 °C min^−1^) of TolA_III_ (○), D418P (•), D418V (▴) and K420P (□) at ~0.14 mg ml^−1^ in 20 mM sodium phosphate, pH 7.0. (d) Table showing derived *T*_m_ from far-UV CD (222 nm) thermal denaturation and SDS sensitivity of TolA_III_ variants (see Fig. S3). (e) Spot test assay showing sensitivity of mutants to ColN (method as in Fig. 2).

The previous NMR data (Hecht *et al.*, 2009) and secondary structure predictions (Fig. 1) indicated that the binding region ColN-T residues S61–F66 (SYHITF) form a β-strand upon binding to TolA. Mutating the (β-strand addition) ColA-binding site on the TolA mutant displayed ColA but not ColN resistance in both spot test (Fig. S2) and liquid culture (data not shown) assays, indicating that this was not the binding site of ColN. As G3p-N1 binds to β3 on the opposite side of TolA_III_, via β-strand addition, and NMR data indicated a common binding site to G3p (Hecht *et al.*, 2009), we then selected this strand for mutation; however, side-chains play a limited role in β-strand addition (Remaut & Waksman, 2006). In the PDB ID: 1Tol structure (Lubkowski *et al.*, 1999), two TolA_III_ β3 residues, D418 and K420, form critical backbone amide–carbonyl hydrogen bonds to G3p-N1 to stabilize the β-sheet interaction between the two proteins. We removed these amide protons by mutation to proline residues which lack the amide protons required for hydrogen bond donation and can only accept hydrogen bonds via the remaining carbonyls. Thus, each proline mutation removed one backbone hydrogen bond which might otherwise stabilize the β-strand addition. These mutations were modelled in the known TolA structure and, following a simple energy minimization, showed, as expected, that prolines had minimal influence on the β-strand backbone conformation (Figs 3a, b). D418V was used as a positive control mutant as valine is a strong β-strand former, can form hydrogen bonds and is one of the ‘allowed’ residues at this position based on the screening work of Karlsson *et al.* (2006). They found that only residues A, V, G and E were both resistant to SDS and sensitive to phage infection.

All three mutants had near WT far-UV CD spectra (Fig. 3c), although the proline mutations did cause a shift of the spectra minima towards 200 nm, which may indicate a small degree of unfolding compared with WT. The structural integrity was also determined by thermal denaturation up to 90 °C using far-UV CD at 222 nm (Fig. 3c, inset). A cooperative unfolding curve was observed in all cases and used to calculate the transition midpoint temperatures (*T*_m_) (Fig. 3d). The WT TolA_III_ had a *T*_m_ of 62.4 °C, as expected (Anderluh *et al.*, 2004). D418P and K420P showed cooperative unfolding curves with reduced *T*_m_ values of 52.9 and 50.3 °C, respectively. Interestingly, the D418V mutant showed both an increased amount of secondary structure, indicated by a more negative minima at 222–208 nm in the far-UV CD, and a higher *T*_m_ than WT (64.1 °C).

Spot test assays (Fig. 3e) and liquid culture killing assays (Fig. S1) showed D418P and K420P were resistant to both ColN and ColA, whereas D418V was as sensitive as WT TolA. Furthermore, D418P and K420P were sensitive to 0.1 % SDS, whilst WT and D418V were not (Figs 3d and S3). This agreed with the known phenotype of mutations within the Tol–Pal system (TolA, TolB, TolQ, TolR and Pal) which caused cells to be hypersensitive to detergents due to disruption of the cell envelope structural integrity (Davies & Reeves, 1975). Finally, both TolA proline mutants were shown to be resistant to ColE9 (Fig. S4), which binds directly to TolB only (Bonsor *et al.*, 2009), and then is bound to TolA via the subsequent interaction of TolB with TolA (Zhang *et al.*, 2010). It is worth noting that this region in *Y. enterocolitica* has the sequence VKFPQ instead of the *E. coli* DFKP, which may allow TolA binding, but not ColN (Weitzel & Larsen, 2008).

### Neither ColN-T nor G3p-N1 binds to TolA_III_ proline mutants

**Fig. 4.**
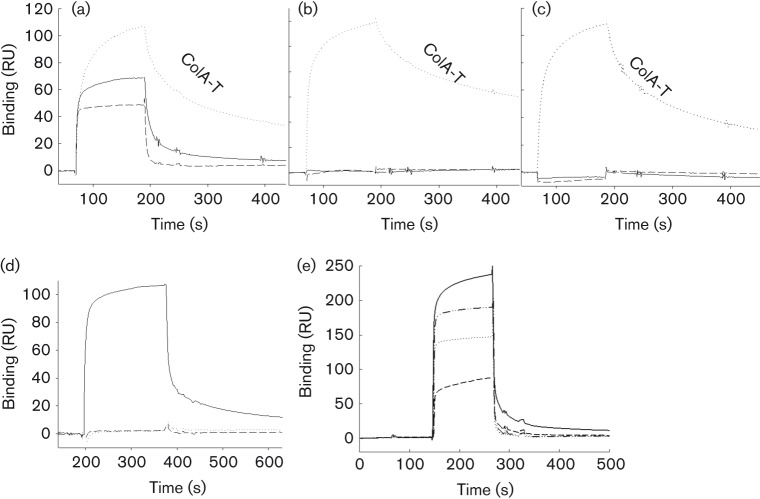
Biacore binding data of ColN-T, ColA-T, G3p-N1 and TolB to WT TolA_III_, D418P and K420P. Around 500 RU of either (a) TolA_III276–421_WT, (b) D418P or (c) K420P protein was immobilized on a CM5 sensor chip using amine coupling. ColN-T (solid line), ColA-T (dotted line) or G3p-N1 (dashed line) (5 µM) were injected over each surface at 30 µl min^−1^ for 120 s followed by regeneration with 10 mM glycine, pH 1.8. (d) TolB (10 µM in HEPES-EP buffer) was injected over the immobilized WT and TolA_III_ mutant surfaces described above at 10 µl min^−1^ for 180 s followed by regeneration with 10 mM glycine, pH 1.8. Only the WT surface bound TolB. (e) WT and mutants of TolB (5 µM) were injected over immobilized TolA_III_. WT TolB (solid line), DGSY-AGAA (dash-dotted line), V101P (dotted line) and VVV-AAA (dashed line). In each case, a control surface which had no immobilized protein was subtracted from the data. Data represent the means of duplicate injections.

For SPR experiments, TolA_III_, D418P and K420P were immobilized on CM5 sensor chips (~550 RU) before ColN-T and G3p-N1 (0.2–50 µM) were injected over the surface (Table 2). Duplicate injections were performed and the signal from a reference (activated/blocked) blank surface was subtracted from each dataset. Our previous work, carried out using glutathione *S*-transferase (GST)–ColN-T fusions binding to immobilized TolA_III_, fitted to a two-site model with *K*_D_ = 0.85 and 0.19 µM for GSTN40 (T domain residues 40–90). In the same study, the results with immobilized GST fusions binding soluble TolA_II–III_ fitted to a one-site model with *K*_D_ = 0.94 µM (Gokce *et al.*, 2000). Thus, the second site was possibly an artefact of TolA immobilization. In the current study, a heterogeneous ligand model fit gave two sites with *K*_D_^1^ = 3.0 µM and *K*_D_^2^ = 4 nM. The *K*_D_^1^ is similar to the *K*_D_ obtained when applying steady-state affinity fitting (1.8 µM) (Fig. S5), a global measure of affinity taking in contributions from both sites, and this is comparable with the published ITC (1 µM) (Raggett *et al.*, 1998), SPR (1.25 µM) (Anderluh *et al.*, 2004) and stopped flow fluorescence (2.3 µM) (Gokce *et al.*, 2000) data. Crucially, no binding to either of the proline mutants occurred. For G3p-N1, we initially followed the previous work of Karlsson *et al.* (2003) immobilizing ~950 RU TolA_III_ on the chip surface, which gave a similar *K*_D_ to the published value (~1 µM); however, the fits were poor due to mass transport effects as indicated by high deviation of the residuals plot and a high χ^2^ (198 RU^2^). Using a lower immobilization level of 550 RU, better fits (χ^2^ = 0.574) were obtained and a 1 : 1 Langmuir binding fit gave a *K*_D_ of 14.9 µM (Fig. S5). G3p-N1 displayed very fast *k*_on_ (2.056×10^4^ s^−1^) and *k*_off_ (0.307 s^−1^) rates. Hence, steady-state affinity is the most accurate measure of *K*_D_, which gave 11.9 µM. No binding was seen by G3p-N1 to either of the proline mutants (Fig. 4).

**Table 2.** SPR data for the interaction of ColN-T, G3p-N1 and G3p-N1 V44P with TolA_III_ and β3 mutants. No binding was noted for G3p-N1 or ColN-T_1–90_ on TolA_III_ D418P or K420P surfaces. No binding was observed for TolA_III_ versus G3p-N1 V44P.

Ligand	Analyte	Binding fits						Steady-state affinity *K*_D_ (µM)
		*k*_a_^1^ (M^−1^ s^−1^)	*k*_d_^1^ (s^−1^)	*k*_a_^2^ (M^−1^ s^−1^)	*k*_d_^2^ (s^−1^)	*K*_D_^1^ (µM)	*K*_D_^2^ (µM)
TolA_III_	G3p-sN1	2.06±0.02×10^4^	0.31±0.002	–	–	15	–	11.9±0.1
TolA_III_	ColN-T	2.22±0.05×10^4^	6.7±0.07×10^−2^	9.67±0.55×10^4^	4.0±0.04×10^−3^	3	0	1.8±0.2

### G3p-N1 V44 is in the TolA_III_-binding site

V44 of G3p-N1 forms two hydrogen bonds with β3 during the β-strand addition to TolA_III_. We mutated this residue to proline to remove one hydrogen bond by removal of the V44 amide proton. Far-UV and near-UV CD spectroscopy showed that the V44P mutant had a similar fold to the WT protein (Fig. S6), and at 288 nm a cooperative thermal unfolding with *T*_m_ 49.6 °C was observed (Fig. S6, inset). The WT G3p-N1 protein had a *T*_m_ of 60.1 °C, similar to the published *T*_m_ (59.8 °C) calculated from the 230 nm transition in far-UV CD by Martin & Schmid (2003). For SPR, TolA_III_ was immobilized on a CM5 sensor chip (~550 RU) and varying concentrations of V44P, up to 250 µM, injected over the surface. A small amount of binding was noted >200 µM (~10 RU), but this was confirmed as non-specific by reversing the ligand/analyte and immobilizing G3p-N1 V44P on the chip surface (488 RU) and passing over TolA_III_ (to 250 µM), where no binding occurred.

#### ColA-T binds to TolA_III_ D418P and K420P.

The interactions of isolated ColN-T, ColA-T and G3p-N1 domains with TolA_III_, D418P and K420P were compared using SPR. Comparable levels of each TolA version were immobilized to the CM5 chip surface. ColN-T, ColA-T and G3p-N1 all bound to the WT TolA_III_ surface (Fig. 4a), but only ColA-T bound to the mutant surface, with ColN-T and G3p-N1 showing no binding (Fig. 4b, c), even at concentrations up to 250 µM (data not shown).

#### TolA D418 and K420 are in the TolB-binding site.

Although ColA-T can bind to TolA_III_ D418P and TolA_III_ K420P (Figs 4b, c), no killing by ColA was seen in spot test assays (Fig. 3e). We believed that this was due to the disruption of TolA–TolB interactions by these mutants. To test this, 10 µM TolB was injected over TolA_III_ WT and mutant surfaces in SPR experiments. Binding was seen on the WT TolA_III_ surface, but not to either of the mutants (Fig. 4d). As the TolA–TolB interaction was thought to be important for Tol function, we tested the outer membrane integrity of *E. coli* cells expressing the TolA point mutants. Extracellular levels of alkaline phosphatase, which is normally trapped in the periplasm, were measured for JC207 ΔTolA cells only and cells complemented with WT TolA_III_, D418P and K420P plasmids. Baseline alkaline phosphatase activity in culture supernatants, measured as OD_410_, was 0.23, whereas the value for JC207 ΔTolA cells was 0.72. For cells complemented with the WT plasmid it was 0.24, but TolA_III_ D418P and K420P gave values of 0.67 and 0.69, respectively. Furthermore, WT complemented cells released approximately one-fifth of the amount of extracellular proteins visible from mutant cultures on 10 % SDS-PAGE (data not shown). Thus, the proline point mutants destabilized the outer membrane and created a clear *tol* phenotype.

### TABS on TolB

A TABS has been proposed in the disordered 12 N-terminal residues of TolB (Bonsor *et al.*, 2009; Carr *et al.*, 2000; Walburger *et al.*, 2002), which thus could also engage in β-strand addition, whilst Dubuisson *et al.* (2002) identified D120 as also being potentially involved in binding. Here, we used alignments of the amino acid sequences of TolB, ColN and G3p-N1 to identify possible TolA_III_-binding motifs (Fig. S4). First, TolB shares the sequence DGSY (109–112) with the core of the ColN-T TABS (59–62) and VVV (99–101) with residues 61–63 of G3p which are central to its TABS β-strand (Fig. S4). To test if these revealed shared binding sequences, we made the following mutations in TolB: DGSY-AGAA, VVV-AAA and V101P (to inhibit backbone hydrogen bonds). DGSY-AGAA and V101P showed a WT far-UV CD spectrum with VVV-AAA showing a more unfolded spectrum (Fig. S7A). Cooperative unfolding curves at 217 nm gave *T*_m_ = 50.9, 52.8 and 50.8 °C for DGSY-AGAA, V101P and VVV-AAA, respectively (data not shown). The *T*_m_ for the WT TolB was 55.4 °C. DGSY-AGAA and V101P mutants displayed WT near-UV CD (Fig. S7B).

TolA_III_ (645 RU) was immobilized on a CM5 chip and 5 µM WT TolB, DGSY-AGAA, V101P or VVV-AAA passed over the surface (Fig. 4e). The 1 : 1 Langmuir binding fits were obtained as described previously (Table 3), giving a *K*_D_ of 37.3 µM for WT TolB binding to TolA_III_, in agreement with the published data of 40 µM obtained using ITC (Bonsor *et al.*, 2009). The DGSY substitution was comparable with WT, but TolB VVV and V101P mutants did not bind TolA.

**Table 3.** SPR data for the interaction of TolA_III_ with TolB WT and mutants

Ligand	Analyte	Binding fits			Steady-state affinity *K*_D_ (µM)
		*k*_a_ (M^−1^ s^−1^)	*k*_d_ (s^−1^)	K_D_ (µM)
TolA_III_	TolB	8.27±0.12×10^3^	0.3±0.004	37	43.2±9.0
DGSY	TolA_III_	2.71±0.05×10^3^	0.10±0.002	39	48.3±4.5
TolA_III_	V101P	4.02±0.21×10^3^	0.59±0.01	146	–
VVV-AAA	TolA_III_	0.33±0.006×10^3^	0.07±0.001	215	–

## Discussion

### ColN-T–TolA_III_ binding

Previous studies (Anderluh *et al.*, 2003, 2004; Gokce *et al.*, 2000; Hecht *et al.*, 2009; Raggett *et al.*, 1998) have defined the TABS of ColN and the structural changes involved upon binding. However, no high-resolution structure is yet available for this complex and the ColN-binding site of TolA_III_ is unknown. TolA has been identified as a translocator for several colicins and phage, with little sequence or structural identity between the proteins involved (Hecht *et al.*, 2009), and we showed that ColN does not bind to TolA_III_ at the recently identified ColA-binding site (Li *et al.*, 2012). It has previously been shown by NMR that the proteins G3p-N1, of filamentous bacteriophage M13, and ColN-T interact with the same region of TolA_III_ (Hecht *et al.*, 2009). Thus, we selected mutant targets using the structure of TolA_III_ complexed with G3p-N1 (PDB ID: 1Tol; Lubkowski *et al.*, 1999) and our previous NMR data (Hecht *et al.*, 2009). Single point mutations along the entire length of TolA α2, and which preserved the native structure, did not affect the toxicity of ColN and ColA. Residues identified by NMR chemical shift changes of >0.55 p.p.m. upon ColN binding (Hecht *et al.*, 2009) were mutated to alanines, but showed little resistance to colicin. Hence, these must be involved in subtle structural rearrangement of TolA_III_ upon binding ColN-T, but the amino acid side-chains are not part of the binding site. The importance of this distinction will become clear below.

### TolA_III_ β-strand addition promiscuity

G3p–TolA (Deprez *et al.*, 2005; Lubkowski *et al.*, 1999), ColA–TolA (Li *et al.*, 2012) and a complex of TolA_III_ of *Vibrio cholerae* with the N-terminal CTXØ minor coat protein pIII of F bacteriophage (CTXØpIII–TolA) (Ford *et al.*, 2012) are all examples of β-strand addition-type mechanisms (Remaut & Waksman, 2006). As only weak sequence constraints determine the β-strand propensity of peptides, promiscuity of binding partners is possible and binders may be either folded or disordered domains. Secondary structure predictions (Cole *et al.*, 2008; Cornilescu *et al.*, 1999; Roy *et al.*, 2010; Zhang, 2008) all suggest that the ColN sequence Y62–H67 adopts a β conformation and, in our previous NMR study, the binding of G3p caused large ^15^N–^1^H-heteronuclear single quantum coherence chemical shift changes in β2 and β3 of TolA. These were largely imitated by ColN-T binding with even larger effects on β3 (Hecht *et al.*, 2009).

Deprez *et al.* (2005) described a ‘stretching’ of the TolA_III_ β-sheet (β2+β1+β3) upon G3p-N1 binding; in particular, β3 is extended and a ‘bulge’ present in β2 disappears. The PDB ID: 1Tol structure shows that residue V44 of G3p contributes to backbone hydrogen bonding in β-addition and we made a G3p V44P mutant to remove the critical amide proton. This mutant did not bind TolA_III_.

Two TolA_III_ residues, D418 and K420 (Figs 1 and 3), are similarly involved in the hydrogen-bonding network with G3p-N1. D418 does show a chemical shift upon ColN-T binding, but was below our 0.55 p.p.m. cutoff for the initial round of mutagenesis, and K420 was not assigned in the NMR spectrum due to its flexibility at the terminus of the protein. Proline mutants should not strongly disrupt the β structure (Chou & Fasman, 1974) and are the clearest way to test for β-strand addition (Fig. 3) (Joseph *et al.*, 2013). Both mutations showed small structural changes by CD and still bound the ColA-T domain in a SPR assay, but showed clear ColN-resistant phenotypes. These results confirm that ColN-T_1–90_ binds via a β-strand addition to TolA_III_ β3 with ColN-T contributing at least one β-strand from within the TolA box, probably residues Y62–H66. Interestingly, Karlsson *et al.* (2006) performed a random mutagenesis screen on this region of TolA_III_, A415–K420, and proline mutants at these positions were screened out as being phage resistant. Their work shows that sequence conservation in this area is second to structural preference with at least one of the β-promoting WT residues (L417, D418 or K420) being retained or replaced with another strong β-former (such as V, L, M and I). It would be interesting to challenge these libraries with ColN and ColA.

### Importance of D418P and K420P mutations in ColA and ColE9 killing

Although the ColN-T- and ColA-T-binding sites are on opposite sides of the TolA_III_ structure, and ColA-T also binds the mutant proteins, the proline mutations within TolA_III_ β3 still cause resistance to ColA. ColA clearly has a different mechanism of translocation to that of ColN and it has been proposed that ColA-T interacts first with TolB in the periplasm via its low-affinity TolB box, followed by high-affinity interaction with TolA (Zhang *et al.*, 2010). TolA has been shown to interact with TolB *in vitro* by ITC (*K*_D_~40 µM) (Bonsor *et al.*, 2009), cross-linking and yeast two-hybrid experiments (Walburger *et al.*, 2002), and this interaction is enhanced (*K*_D_~13 µM) when ColE9 binds to TolB (Bonsor *et al.*, 2009).

However, the interpretation of ColA and ColE9 activity is complicated by the discovery that these point mutations give cells a ΔTol phenotype including sensitivity to SDS. This implies a disruption of the cell envelope due to the inhibition of the TolA–TolB complex. We used an alkaline phosphatase assay (Lazzaroni & Portalier, 1981; Torriani, 1966) to show that periplasmic alkaline phosphatase, which is larger (79 kDa) than TolB (47 kDa), leaches into the extracellular medium of ΔTolA periplasmic-leaky cells (JC207). Crucially, similar amounts of alkaline phosphatase leached out from TolA_III_ D418 and K420 mutants. As we cannot determine any effects on the TolB–Pal interaction, we cannot thus rule out that the inability of ColA and ColE9 to kill these mutants may be due to very low levels of periplasmic TolB rather than a need for a TolA–TolB interaction to maintain the ColA–TolB–TolA_III_ or ColE9–TolB–TolA_III_ complex.

The two different binding sites for TolB and ColA-T on TolA_III_ support the formation *in vivo* of a heterotrimeric complex which has been observed by gel filtration *in vitro* (Hecht *et al.*, 2010), but it is not clear if a direct TolB–TolA interaction is required for the toxic activity of ColA, as it is for ColE9, or whether the bifunctional ColA-T cross-links the complex. The disordered extreme N terminus of TolB has been shown to bind TolA_III_ and the NMR spectrum of a TolA_III_–TolB^1–13^ complex resembles that of a TolA_III_–ColN-T complex (Li *et al.*, 2012). Thus it is likely that TolB and ColN-T bind via a similar β-strand addition mechanism.

The combined data for three different Group A (Tol-dependent) colicins give an insight into their use of the same Tol proteins in fundamentally different ways, but for apparently the same purpose. ColE9 binds only TolB, but by doing so stabilizes the TolA–TolB complex (Bonsor *et al.*, 2009). ColA requires direct interaction with both TolA and TolB, but it is not clear if a direct TolA–TolB complex is required (Penfold *et al.*, 2012). Finally, ColN is unique in not requiring TolB and binds TolA at the precise site where TolB would bind. ColN consistently displays minimalistic behaviour as demonstrated by its small size, LPS receptor (Johnson *et al.*, 2014), single protein receptor (Clifton *et al.*, 2012; Johnson *et al.*, 2013) and lack of TolB dependence. This simplicity makes ColN unique in colicin biology, and the disruption of TolA–TolB may be relevant to our recent discovery that ColN-T alone can target and kill *E. coli* (Johnson *et al.*, 2013).
